# The paradigm shift: re-evaluating preclinical animal models for colorectal cancer in the precision medicine era

**DOI:** 10.3389/fimmu.2025.1744692

**Published:** 2026-01-15

**Authors:** Qin Huang, Shucan Wei, Jiahui Yu, Yancen Wu, Hao Lai, Wene Wei, Linhai Yan, Chenlin Su, Wei Shi, Zijie Su

**Affiliations:** 1 Department of Experimental Research, Guangxi Key Laboratory of Basic and Translational Research for Colorectal Cancer, Guangxi Medical University Cancer Hospital, Nanning, China; 2 Department of Colorectal Surgery, Guangxi Clinical Research Center for Colorectal Cancer, Guangxi Medical University Cancer Hospital, Nanning, China; 3 Department of Laboratory Animal Science, Laboratory Animal Center, Guangxi Medical University, Nanning, China; 4 Guangxi Engineering Research Center of China-ASEAN Laboratory Animal Science and Innovation, Guangxi Medical University, Nanning, China

**Keywords:** chemical carcinogen, colorectal cancer, patient-derived xenograft, precision medicine, transgene animal

## Abstract

Colorectal cancer (CRC) remains a major global health burden. While precision therapies like anti-PD-1 and anti-EGFR antibodies show remarkable efficacy, their application is constrained by stringent biomarker requirements, limiting patient benefit. Diverse animal models—including chemically induced, genetically engineered, and transplantation-based systems—have advanced our understanding of CRC pathogenesis but exhibit limited power in predicting therapeutic outcomes for defined patient subgroups. A central challenge is their imperfect recapitulation of key aspects of human CRC biology, specifically anatomical tumor localization, faithful representation of the tumor immune microenvironment (TME), and a frequent lack of rigorous molecular characterization. This gap underscores the urgent need for advanced models that better mirror human disease to support translational research. This review critically evaluates the establishment, advantages, and limitations of prevalent CRC models, focusing on their capacity to replicate key immunological features of human CRC, such as the complex immune landscape and response to immunotherapies. We examine how discrepancies in anatomical site, immune cell composition, and host immunity between animal models and human patients compromise predictive accuracy, particularly for evaluating immune-checkpoint inhibitors (ICIs) in microsatellite-stable (MSS) tumors. By synthesizing these critiques, we aim to provide a framework for developing immunologically relevant models to accelerate the discovery of effective, personalized immunotherapies for CRC.

## 1 Introduction

Colorectal cancer (CRC) is a major global public health challenge, ranking as the third most prevalent malignancy and the second leading cause of cancer-related mortality worldwide (1). The emergence of precision oncology has begun to transform cancer treatment, moving beyond a one-size-fits-all approach to embrace therapies tailored to the genetic and molecular profile of an individual’s tumor. This paradigm shift is exemplified by the success of targeted agents in molecularly defined subsets of cancers, such as immune checkpoint inhibitors in mismatch repair-deficient (dMMR) CRC and anti-EGFR antibodies in wild-type RAS tumors. However, the broader application of these therapies in CRC is often hampered by intrinsic and acquired resistance, tumor heterogeneity, and the lack of predictive biomarkers for many emerging agents.

**Figure 1:**
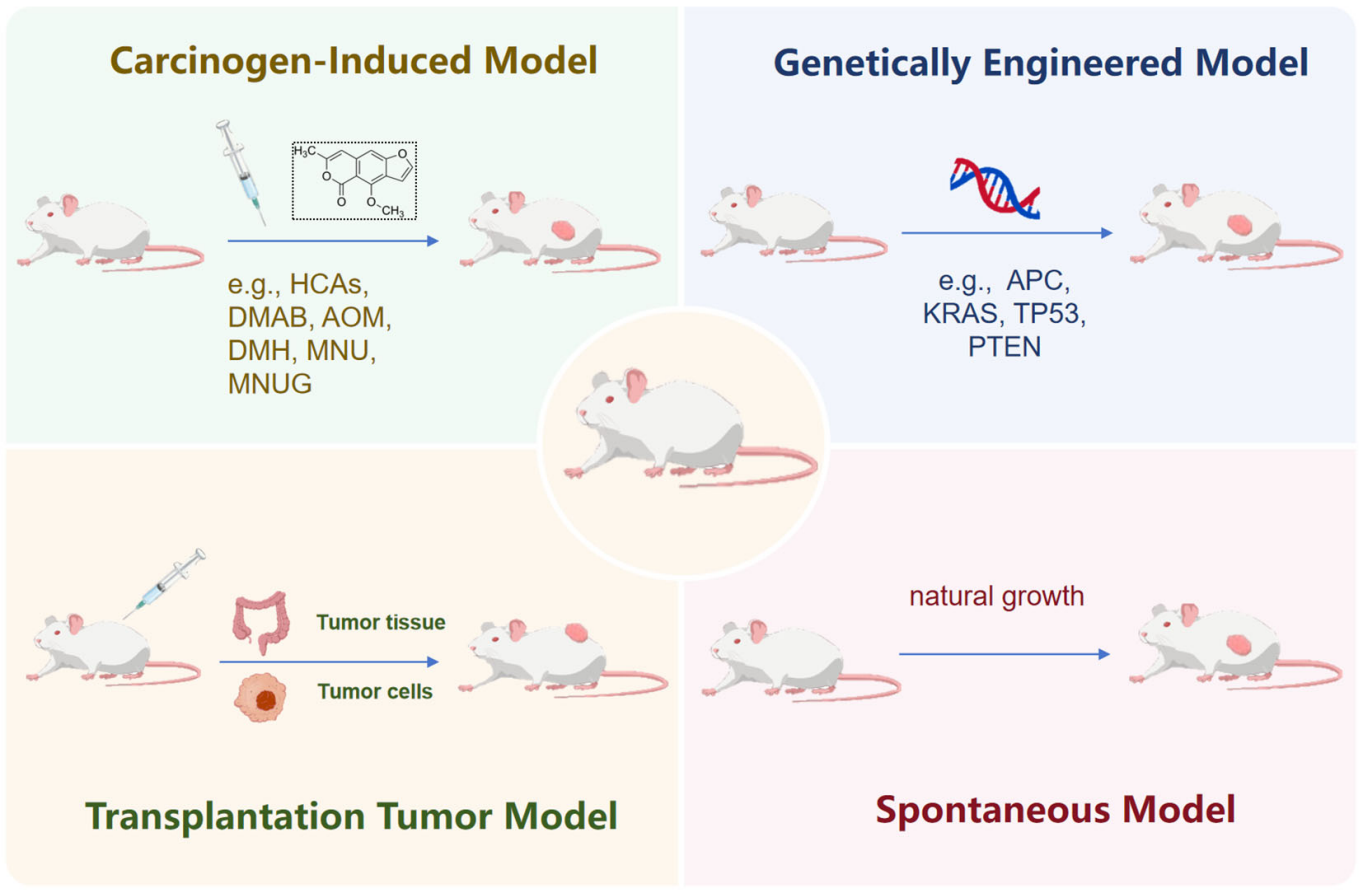
Construction strategies for four classic mouse tumor models. This schematic illustrates the key approaches to generating chemically induced, genetically engineered, transplantation-based, and spontaneous tumor models, highlighting their distinct methodologies and applications in cancer research.

The successful translation of novel therapeutic concepts from bench to bedside is critically dependent on preclinical models that faithfully recapitulate the complexity of human disease. Traditional CRC animal models, including chemically induced (*e.g.*, AOM/DSS), genetically engineered (*e.g.*, APC^Min/+^mice), and transplantation-based (*e.g.*, CDX, PDX) systems, have provided invaluable insights into carcinogenesis pathways and initial drug efficacy screening (**Figure 1**). Yet, these models often fall short in mirroring the nuanced tumor microenvironment (TME), the interplay with the immune system, and the specific clinical scenarios of human CRC, particularly in the context of precision oncology. For instance, while the APC^Min/+^ mouse has been instrumental in elucidating the Wnt pathway, its predominant development of small intestinal tumors limits its translational relevance to human colorectal cancer, which arises in a distinct anatomical and immunological context. This discrepancy highlights a significant gap in our preclinical toolbox—the lack of models that can accurately predict patient-specific responses to increasingly targeted therapies.

Furthermore, the rising incidence of CRC associated with modern lifestyle factors, such as high-fat diets and dysbiosis of the gut microbiota, necessitates the development of models that incorporate these environmental and microbiome influences to study inflammation- and metabolism-driven tumor genesis. The limitations of current models become particularly apparent when attempting to evaluate combination therapies, assess immune-modulating agents, or model the metastatic cascade—all key areas in modern drug development.

This review seeks to critically evaluate the current landscape of laboratory animal models for CRC through the lens of precision medicine. We will first detail the foundational principles, construction methods, and applications of classical model systems. We will then specifically examine emerging models designed to study the role of critical risk factors, including chronic inflammation, dietary components, and specific microbial pathogens. A central focus of our discussion will be a critical appraisal of the advantages and inherent limitations of each model system in addressing the specific challenges of precision oncology, such as modeling tumor heterogeneity, predicting immunotherapy responses, and facilitating the study of rare molecular subtypes. Finally, we will discuss the persistent hurdles in modeling the human CRC immune landscape and disease progression, and explore future directions aimed at refining these essential preclinical tools to better guide therapeutic decisions and improve clinical outcomes.

## 2 Precision therapy for CRC: current status

Precision therapy is now a cornerstone in the management of CRC management, fundamentally relying on molecular subtyping to define tumor biology and uncover therapeutic targets. Current classification systems are primarily based on three key oncogenic pathways: chromosomal instability (CIN, ~85% prevalence), microsatellite instability (MSI, ~15% prevalence), and CpG island methylator phenotype (CIMP, ~22% prevalence), along with their associated driver mutations (2, 3). This molecular framework underpins individualized treatment strategies.

The molecular subtyping of CRC relies on distinct detection methodologies for each classification:

CIN is assessed through techniques that evaluate large-scale genomic alterations. Karyotyping provides a cytogenetic overview but offers lower resolution (5–10 Mb). For higher resolution screening of copy number variations, single nucleotide polymorphism (SNP) arrays (100–500 kb) are commonly employed (4). Ultimately, whole-genome sequencing (WGS) offers the most precise characterization (<100 kb resolution), enabling the detailed mapping of specific amplification/deletion sites and the determination of their frequencies in tumors suspected of having a high CIN burden (5).

MSI status is primarily determined by analyzing short, repetitive DNA sequences. The standard method involves polymerase chain reaction (PCR) amplification of a classic panel of five mononucleotide and dinucleotide markers (BAT25, BAT26, NR-21, NR-22, and NR-24) (6). Instability in two or more loci defines a tumor as MSI-High (MSI-H). Alternatively, immunohistochemistry (IHC) for the four mismatch repair (MMR) proteins (MLH1, MSH2, MSH6, PMS2) serves as a reliable surrogate; loss of nuclear expression for any of these proteins indicates dMMR, which shows 90-95% concordance with MSI-H status. In cases of discordant results, sequencing-based verification is recommended. For instance, absent MLH1 expression by IHC is often associated with promoter hypermethylation, a finding that warrants confirmation via direct assessment of microsatellite status.

CIMP, defined by a high degree of promoter CpG island methylation, is assessed using various detection methods range from targeted assays, such as methylation-specific PCR (MSP) and quantitative pyrosequencing, to comprehensive genome-wide methylation sequencing. In clinical practice, pyrosequencing is frequently used to quantify methylation levels at specific loci, such as the MLH1promoter. This distinction is critical for guiding therapy, as it helps distinguish sporadic MSI-H tumors (often MLH1-methylated and CIMP-high) from those associated with Lynch syndrome (7).

Several biomarkers critically guide clinical decision-making. RAS mutations (~40% prevalence) determine eligibility for anti-EGFR therapies (*e.g.*, cetuximab), effective only in wild-type cases (8). The BRAF V600E mutation (< 10% prevalence), associated with poor prognosis, typically requires targeted combination regimens (encorafenib combined with cetuximab) (9). MSI-H/dMMR tumors (~15% prevalence) demonstrate exceptional responsiveness to immune checkpoint inhibitors like pembrolizumab, while MSS tumors remain largely resistant (10). For patients with HER2 amplification (2-3% prevalence), trastuzumab-based regimens show efficacy (11). Those with rare NTRK fusions (0.2-2.4% prevalence) benefit from TRK inhibitors such as larotrectinib and entrectinib (12).

Despite guideline recommendations for universal molecular profiling, its implementation remains suboptimal. Testing rates reported as low as 20-30% in some countries and regions, including China, largely due to logistical and educational barriers (13). Beyond limited testing access, a more profound challenge is the marked interpatient heterogeneity in treatment response. Many patients either lack targetable alterations or develop resistance to targeted therapies. Consequently, a substantial proportion cannot benefit from current precision strategies. This dual challenge-limited access to molecular profiling and the inherent limitations of existing targeted agents-highlights a critical gap between the theoretical potential of precision oncology and its real-world clinical application. Thus, robust preclinical CRC models that faithfully recapitulate human disease biology are urgently needed to validate biomarker-driven therapeutic strategies, understanding resistance mechanisms, and developing effective treatments for a broader patient population.

## 3 Current animal models in CRC research

### 3.1 Carcinogen-induced models

Carcinogen-induced models (CIMs) are widely used in CRC research to simulate tumor development under controlled conditions (14). These models employ chemical carcinogens administered via oral, intraperitoneal/subcutaneous/intramuscular injection, or rectal routes to induce malignant transformations in the intestinal epithelium (15). CIMs are valuable for elucidating molecular pathways and identifying potential therapeutic targets (16). Commonly used compounds including: heterocyclic amines (*e.g*., PhIP), aromatic amines (*e.g*., DMAB), alkylating agents (*e.g*., MNU, MNNG), and dimethylhydrazine derivatives (*e.g*., DMH, AOM) (15, 17). These carcinogens are classified as either indirect-acting (requiring metabolic activation, such as DMAB and AOM) or direct-acting (such as MNU and MNNG) agents (**Table 1**).

**Table 1:** Comparative characterization of chemically-induced CRC models.

Types	Compound	Reactive form	Combine factors	Gene mutation	MSI status
HCAs	IQ, PhIP,	N-OH-PhIP	DSS,HFD	APC, KRAS, CTNNB1 (172)	MSI, MMR (173)
Aromatic amines	DMAB	nitreniumion intermediates	HFD	APC, KRAS (174)	Unknown
Alkyl nitrosamine compounds	MNU, MNNG	Unrequired	Not clearly defined	KRAS, APC (173)	MSI (173)
Dimethylhydrazine and azoxymethane	DMH, AOM	MAM	DSS, APC^Min/+^	KRAS (6%), APC, CTNNB1 (175)	MSI-L (176)

The MSI status of some models varies due to different experimental conditions (such as carcinogen dose, animal strain), and has not been verified by multiple centers.

AOM, azoxymethane; DMAB, 3,2’-dimethyl-4-aminobiphenyl; DMH, 1,2-dimethylhydrazine; DSS, dextran sodium sulfate; HCAs, Heterocyclic amines; HFD, high-fat diet; IQ, 2-amino-3-methylimidazo[4,5-f]quinoline; MAM, methylazoxymethanol; MNU, N-methyl-N-nitrosourea; MNNG, N-methyl-N’-nitro-N-nitrosoguanidine; MSI, Microsatellite instability; MSI-L, Microsatellite instability-low; PhIP, 2-amino-1-methyl-6-phenylimidazo[4,5-b]pyridine.

Among indirect-acting agents, PhIP combined with dextran sulfate sodium (DSS) promotes inflammation-driven carcinogenesis, mimicking human sporadic CRC and enabling studies on DNA damage and prevention (18, 19). DMAB requires metabolic activation to generate reactive intermediates that foster tumor formation from ROS-induced inflammation, and it is often enhanced by high-fat diets (15). In contrast, MNU and MNNG mutations via rectal administration, yielding short tumor latency but requiring technical expertise (20) (15, 21). AOM, a procarcinogen activated through hepatic metabolism, induces point mutations (*e.g*., O^6^-methylguanine lesions) and, when combined with DSS, accelerates tumorigenesis by synergizing genetic mutations with inflammatory microenvironments (*e.g*., NF-κB activation), closely mimicking human colitis-associated CRC (22).

These models exhibit genetic and pathological similarities to human CRC but face challenges such as prolonged experimental durations due to stochastic tumor development and variability influenced by rodent sex, age, genetic background, gut microbiota, and immune conditions (16). The CIMs tumors are mainly concentrated in the distal part of the colon and rectum, which differs from the widespread distribution of human colorectal cancer (with a high incidence in the rectum and sigmoid colon); the driving mutations are single, the incidence of MSI-H is low (5%-10%), and the molecular subtype matching is poor; the response prediction of EGFR inhibitors and immune checkpoint inhibitors is disconnected from clinical practice, which is prone to cause treatment failure (23, 24). These differences may be related to the induction method of the model, intestinal physiology, as well as human lifestyle and environmental factors.

CIM, represented by AOM/DSS model, has obvious shortcomings compared with humanized model in simulating TME. Its immune cells are all derived from the mouse host, and it is difficult to reproduce the infiltration pattern of CD8^+^T cells, the proportion of regulatory T cells and the secretion of IFN-γ and IL-10 in CRC TME (25). The matching degree of immunosuppressive cell components to human is less than 30%, and it is difficult to reproduce the immunosuppressive TME of MSS tumors. Specifically, the composition of T cells, immunosuppressive markers and the degree of infiltration of related cells were significantly different from the TME of human MSS colorectal cancer. At the same time, due to the random effects of carcinogens and the differences in host immune status, the interindividual TME variability is large, and it is difficult to dynamically monitor immune-related indicators. However, the humanized model can reconstruct the human immune cell system in mice and reproduce the complex cellular and cytokine characteristics of human CRC TME. This further highlights the application limitations of the CIM model. Despite these limitations, CIMs provide critical insights into inflammation-cancer transitions and subtype-specific drug screening. For example, AOM/DSS models recapitulate “inflammation-driven” (CMS4-like) and “Wnt-driven” (CMS2-like) subtypes, facilitating the evaluation of targeted therapies like iNOS or KRAS inhibitors (26, 27). In the PhIP/DSS and DMAB models, PD-1 inhibitors, cetuximab, celecoxib, and CD73 inhibitors also exert targeted therapeutic effects (28–30).

Regarding the response mechanism of the AOM/DSS model to PD-1 inhibitors, it is hypothesized to involve inflammation-driven upregulation of PD-L1, potentially via NF-κB pathway activation, which can increase PD-L1 transcription levels by approximately 2.5 to 3-fold (31). Although the MSI status in this model has not been unequivocally validated in certain studies, this mechanism fundamentally differs from the immune response triggered by high tumor mutational burden characteristic of human MSI-H tumors. In contrast, patient-derived xenograft (PDX) models, which retain the molecular subtypes and genetic mutations of the original patient tumors, have demonstrated utility in predicting responses to various targeted therapies. This includes forecasting the efficacy of EGFR-targeted treatments, BRAF inhibitor-based combination regimens, and PARP inhibitors, thereby offering valuable guidance for therapeutic decision-making.

### 3.2 Genetically engineered models

Genetically engineered mouse (GEM) models represent a sophisticated approach to studying CRC pathogenesis by introducing targeted modifications in key driver genes, thereby enabling the investigation of spontaneous tumorigenesis with high genetic stability (32). These models effectively recapitulate significant genetic alterations associated with both sporadic and inherited forms of CRC, facilitating the exploration of gene-environment interactions (33) and molecular mechanisms underlying disease progression (34).

Among the most pivotal GEM mice are those targeting the APC gene, a key element involving multiple cellular processes (*e.g*., β-catenin degradation, cytoskeletal organization, cell cycle progression, apoptosis, and cell adhesion) (35), as well as a critical tumor suppressor regulating Wnt/β-catenin signaling (36). The classic APC^Min/+^ mouse model develops numerous intestinal adenomas within 15 weeks and increases tumor burden when combining the KRAS activation, mimicking human familial adenomatous polyposis (FAP) (37, 38).

Some GEM models (such as APC deletion combined with KRAS mutation) can replicate the tumor immunosuppressive TME of MSS, but the defects are significant: TME is a mouse immune environment, lacks the immune regulatory network of human intestinal cancer flora, and the dendritic cells are not mature enough, which affects the evaluation of immune drug efficacy 70% of human CRC occurs in the colon and rectum, while most GEM tumors are located in the small intestine. The anatomical differences prevent GEM models from reproducing key features of human distal CRC and overestimate the efficacy of combination therapy. Rather than conventional models where spontaneous tumors primarily localize to the small intestine, tissue-specific biallelic knockout models exhibit colonic tumors with improved anatomical relevance, for examples, APC^flox/flox^ mice harboring nonsense mutations at specific codons (**Table 2**) (*e.g.*, T850, T242, T716, or T1638) crossing with Cre^+^ mice (Villin-Cre or CDX2-Cre) (39, 40). However, these models face limitations such as predominant small intestinal tumor localization, rare metastasis, and prolonged breeding timelines, as well as oversimplification of human tumor complexity through single-gene modifications.

**Table 2:** The phenotypic disparity in tumor spectrum between APC mutant mouse models and human CRC.

Model	Truncated mutation site	Number of tumors	Location of tumor	References
APC^Δ242/+^	aa 242	177 polyps	Mainly in small intestine	(177, 178)
APC^Δ474/+^	aa 474	122 polyps	Mainly in small intestine, few in colon and stomach	(177–179)
APC^580/+^	aa 580	120 polyps	Mostly in the distal rectum	(180, 181)
APC^Δ14/+^	aa 580	36 polyps	Mainly in small intestine	(178, 179)
Apc^Δ15/+^	aa 650	185 polyps	Mostly in the small intestine	(182)
APC^Δ716/+^	aa 716	58–256 polyps	Mainly in small intestine	(183, 184)
APC^Min/+^	aa 850	20–100 polyps	60% in small intestine, few in colon and very few in stomach	(38, 185)
APC^Δ1061/+^	aa 1061	Unknown	Mainly in small intestine	(186, 187)
APC^Δ1309/+^	aa 1309	33–37 polyps	Mainly in small intestine, few in stomach and colon	(188–190)
APC^1322T/+^	aa 1322	200 polyps	Most in small intestine, few in colon and stomach	(191, 192)
APC^1638N/+^	aa 1638	<10 polyps	In small intestine, colon and stomach	(193)

TP53-mutant models, for examples, APC^Min/+^p53^−/−^ or APC^Δ716+^Trp53 R270H, demonstrate enhanced tumor malignancy and accelerated adenoma-to-carcinoma progression, though they require compound genetic alterations and exhibit variable penetrance (41). KRAS-mutant models, frequently combined with APC loss (*e.g.*, APC^Min/+^ KRAS^G12D^), recapitulate the adenoma-carcinoma sequence and are valuable for studying advanced disease and combination therapies (41, 42). Similarly, GEM models targeting the PI3K/PTEN pathway (*e.g*., APC^Min/+^ PTEN^+/−^) (43) or the TGF-β/SMAD signaling (44), highlight roles in tumor invasion, metastasis, and therapy resistance, but often depend on synergistic mutations for robust phenotypes. In BRAF V600E mutation-driven models (*e.g.*, Vil-Cre; Braf^V637E/+^; p53^LSL-R172H/+^ mice), tumor metastasis to local lymph nodes, pancreas or lungs occasionally develops, with an incidence ranging from 12% to 25% (45). These models replicate alternative serrated pathway activation and metastatic potential (46), yet still require multiple genetic alterations.

A significant immunological limitation of GEM models is their murine-derived immune system, which lacks human MHC molecules (such as HLA-A and HLA-B). This prevents the simulation of human tumor antigen presentation. Common tumor antigens in human CRC (such as CEA and MUC1) need to be presented through HLA-A to activate CD8^+^ T cells, but murine MHC molecules cannot effectively bind human antigen peptides, leading to differences in T cell activation efficiency between the model and humans (47). In addition, the T cell receptor (TCR) repertoire of murine origin is significantly different from that of humans (48, 49). The diversity of the human TCR repertoire is about 10^12^, while that of murine origin is only 10^8^, resulting in insufficient diversity of immune responses in the model and inability to simulate the complex antigen-specific T cell responses in human CRC.

These GEM models have become indispensable for precision oncology research of CRC. In response to APC mutation, the APC^Min/+^ model has validated PRAP inhibitor in modulating immunosuppressive pathways and enhancing anti-PD-1 efficacy in colon cancer treatments (50, 51). KRAS-driven models demonstrate the efficacy of MEK inhibitors and their synergy with immunotherapy (52). Compound models like the APC/KRAS/Trp53 triple mutation (AKP) reveal the so-called “synthetic lethality” effect with PARP inhibition and chemotherapy (53, 54). BRAF V600E mutation models support combined targeting of MAPK and immune pathways to overcome resistance (55–57), while in TGF-β receptor inhibitor may suppress tumor metastasis in the SMAD4-deficient high-metastatic model. These models provide more efficient tool for the research of high-risk CRC (58, 59).

The GEM model has significant limitations in the preclinical research of human CRC: over 70% of human CRC occurs in the distal colon (with a high BRAF mutation rate and a more immunosuppressive tumor microenvironment), while the classic GEM model has over 80% of tumors concentrated in the small intestine. There are significant differences in anatomy, microbiota, immunity, and gene expression between the two. Specifically, the immune simulation bias of the GEM model is obvious, with T-cell receptor diversity being only one-tenth of that in humans, MHC homology being lower than 50%, and the lack of the co-evolution process between the human microbiota and the immune system. The molecular subtypes are not fully covered, only including the CMS/4 subtype, while missing key subtypes such as MSI-H; the accuracy of efficacy prediction is poor, with the response rate to EGFR inhibitors being much higher than that in clinical trials, seriously interfering with the evaluation of targeted therapy and immunotherapy (48). Although GEM model cannot precisely replicate the pathogenesis mechanism of distant CRC, it needs to be optimized through combining patient-derived organoids (PDO), humanized mice and other models. However, it also has unique advantages--it can precisely simulate the molecular subtypes of CRC, identify specific mutation treatment targets and verify targeted treatment plans. By leveraging the “gene mutation - targeted drug - effect verification” system, it closely connects experimental research with clinical translation.

### 3.3 Transplantation models of CRC

Transplantation models, which involve implanting human or rodent tumor tissues or cells into immunodeficient or immunocompetent mice, are widely used due to their rapid tumor formation, high reproducibility, and cost-effectiveness. These models are invaluable for large-scale drug screening and tumor microenvironment studies (60). They can be classified based on implant type (cell, organ, or tumor fragment), transplantation site (orthotopic or ectopic), and host type (allogeneic or xenogeneic) (61, 62).

Common immunodeficient hosts include nude mice, NOD/SCID, NSG, and inbred strains like BALB/c (63, 64). The nine commonly used CRC cell lines—Caco-2, HT29, SW480, SW620, DLD-1, HCT116, LoVo, LS174T, RKO—collectively cover the main molecular subtypes of CRC (**Table 3**). Cell lines are selected to reflect CRC molecular heterogeneity: MSS lines (*e.g.*, SW480/HT-29) simulate microsatellite-stable subtypes; MSI-H lines (*e.g.*, HCT116/LoVo) model instability subtypes; angiogenesis-associated lines (*e.g.*, LS174T) study vascular mechanisms; and BRAF-mutant lines (*e.g.*, Colo205) target V600E-driven CRC (65, 66). Key approaches include subcutaneous injection for tumor growth monitoring, orthotopic implantation for invasion/metastasis studies, and intraperitoneal injection for modeling peritoneal metastasis, and evaluation of anti-metastatic therapies (67, 68).

**Table 3:** Molecular and phenotypic characterization of CRC cell lines for precision medicine research.

Cell lines	MSI status	CMS	Mutated genes	Applications in precision medicine	References
Caco-2	MSS	CMS2/3	APC, TP53, SMAD4, CTNNB1	Drug absorption, EGFR sensitivity, and studies on epithelial barrier, differentiation and drug transport absorption.	(194, 195)
HT29	MSS	CMS2/4	APC, BRAF^V600E^,TP53	BRAF therapy, EGFR resistance, gastrointestinal barrier research, CIN and MAPK activation.	(196, 197)
SW480	MSS	CMS2/3	KRAS^G12V^, APC, TP53	KRAS research, research on oxaliplatin resistance.	(198, 199)
SW620	MSS	CMS4	KRAS, TP53, EGFR	Modeling of metastatic colorectal cancer, aspects related to oxidative stress and Wnt dependence in advanced diseases.	(200, 201)
DLD-1	MSI-H	CMS1	KRAS^G13D^, PIK3CA, TP53, APC	PI3K, MSI-H drug resistance and immune evasion; exploration of related pathway mechanisms and drug/escape mechanisms.	(202, 203)
HCT116	MSI-H	CMS1	KRAS^G13D^, CTNNB1, TGFBR2	Functional studies of KRAS, PI3K, MSI-H and p53; the relationship between MMR deficiency and tumor occurrence triggered by KRAS; DNA repair and proliferation.	(204, 205)
LoVo	MSI-H	CMS1	KRAS^G13D^, APC, TGFBR2, TP53 WT	Immune escape, MSI-H therapy, and metastasis model research.	(206, 207)
LS174T	MSI-H	CMS1	KRAS^G12D^, CTNNB1, PIK3CA	Mucin-targeted therapy, MSI-H research, mucin biology and CMS1-like CRC phenotype study.	(194)
RKO	MSI-H	CMS1	BRAF, MLH1	MSI-H tumor study: linking metabolic stress and replication stress.	(200)

These models are tailored to specific CRC molecular subtypes in precision medicine. MSS subtypes use cell lines like SW480 to assess the efficacy of anti-angiogenic therapies (*e.g.*, bevacizumab) or combination chemotherapies (69). MSI-H models (*e.g.*, HCT116) assess immune checkpoint inhibitors (*e.g.*, anti-PD-1 antibodies), leveraging their high tumor mutational burden and enhanced immunotherapy sensitivity (70). Angiogenesis-associated subtypes employ lines like LS174T) to screen angiogenesis inhibitors (*e.g.*, sunitinib) (71), while BRAF-mutant models (*e.g.*, Colo205) test BRAF inhibitors (*e.g.*, vemurafenib) and its combination strategies (72).

**Figure 2:**
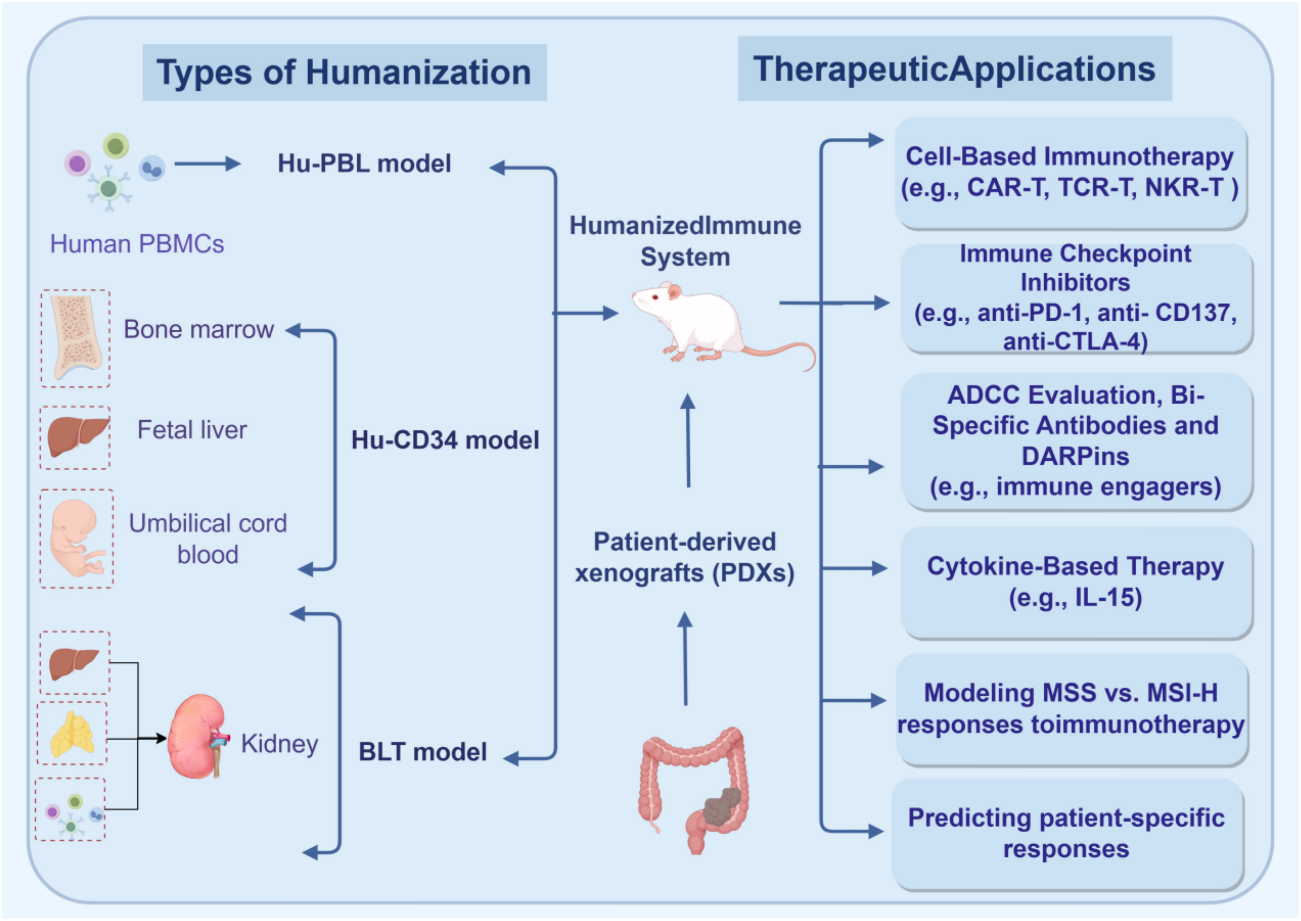
Schematic overview of humanized mouse models and their therapeutic applications in cancer immunotherapy. Immunodeficient mice are humanized through three primary approaches: (1) engraftment of human PBMCs to generate the human peripheral blood leukocyte (Hu-PBL) model; (2) transplantation of CD34^+^ hematopoietic stem cells (from sources such as bone marrow, fetal liver, or umbilical cord blood) to create the human CD34^+^ (Hu-CD34) model; and (3) combined implantation of fetal liver, thymus fragments (under the renal capsule), and matched hematopoietic stem cells to form the bone-liver-thymus (BLT) model. These humanized systems, when combined with PDXs, enable the study of human tumor-immune interactions and support diverse therapeutic applications, including: (1) testing cell-based immunotherapies (e.g., CAR-T, TCR-T), (2) evaluating immune checkpoint inhibitors (e.g., anti-PD-1, anti-CTLA-4), (3) assessing ADCC, bispecific antibodies, and DARPins, (4) investigating cytokine-based therapies (e.g., IL-15), (5) modeling differential responses to immunotherapy in MSS vs. MSI-H tumors, and (6) predicting patient-specific therapeutic responses.(Created with Figdraw.com). CAR-T, Chimeric Antigen Receptor T cells; TCR-T, T Cell Receptor-Engineered T cells; CTLA-4, Cytotoxic T-Lymphocyte-Associated Protein 4; ADCC, Antibody-Dependent Cellular Cytotoxicity; DARPins, Designed Ankyrin Repeat Proteins; IL-15, Interleukin-15; MSS, Microsatellite Stable; MSI-H, Microsatellite Instability-High.

Humanized mouse models, often utilizing NSG strains, are engineered to constitute a human immune system via CD34^+^ hematopoietic stem cells or peripheral blood mononuclear cells (PBMCs), enabling co-transplantation with PDX models (73–75). These systems simulate human-specific immune processes, providing a platform for immune-related research, especially for poorly immunogenic subtypes like MSS (76, 77). They replicate clinical responses to immunotherapy (*e.g.*, different PD-1 inhibitor effects in MSI-H against MSS tumors) and support the evaluation of novel therapies like CAR-T cells and bispecific antibodies, aiding immune biomarker identification and immune crosstalk analysis (78–80). However, limitations include: (1) complex immune system reconstruction (2), high costs, (3) prolonged experimental cycles, (4) incomplete reconstruction efficiency, (5) graft-versus-host disease (GVHD) risks in PBMC models (4–5 week experimental window), (6) murine stromal influence, and (7) ethical/sample scalability concerns (81, 82). Autologous humanized models use patient-matched immune and tumor tissues to predict immunotherapy efficacy and test combination therapies (*e.g.*, cytokines with checkpoint inhibitors) for resistant “cold” tumors (**Figure 2**) (83). Humanized mouse models also have key shortcomings that limit their application in immunotherapy evaluation: 1) Low immune reconstitution efficiency: The chimerism rate of human immune cells after CD34^+^ hematopoietic stem cell transplantation is usually only 30%-50%, and T cell maturation is insufficient, lacking immune memory function; 2) Abnormal cytokine signaling: Cytokines such as IL-2 and IFN-γ secreted by human immune cells cannot effectively activate murine stromal cells, leading to abnormal immune-stromal interactions; 3) Murine myeloid cell bias: Murine macrophages and dendritic cells still dominate in humanized models (60%-70%), affecting the authenticity of immune response evaluation (84, 85).

Transplantation models closely mimic clinical tumor characteristics and molecular heterogeneity of patients, serving as key platforms for precision treatment research. They replicate the natural TME to evaluate anti-tumor and anti-metastatic efficacy of agents like Wnt inhibitors and anti-angiogenic drugs (86). The issue of immune rejection in transplantation models can be circumvented by using severely immunodeficient mice such as NSG (lacking T, B, and NK cells), but the interaction between murine stromal cells and human tumor cells may still influence the response to immunotherapy. For example, CXCL12 secreted by murine fibroblasts can promote PD-L1 expression in tumor cells (upregulated ~1.8-fold) (87).

Cell-line-derived xenograft (CDX) models rapidly screen drugs, confirming the specificity of BRAF inhibitors and revealing mechanisms of new AKT inhibitors like costunolide (CTD) (88, 89). PDX retain molecular subtypes and gene mutations, predicting the efficacy of EGFR-targeted therapy, BRAF inhibitor combination regimens (*e.g.*, dasatinib), and PARP inhibitors to guide treatment decisions (89–91). Although murine mesenchyme retains some tumor-cell characteristics, secreted murine-specific cytokines such as murine IL-6 alter human tumor-cell signaling. Most myeloid cells are derived from mice, forming “mouse myeloid bias”, which is not conducive to simulating human immunosuppressive TME (92). HER2 antibody-drug conjugates exhibit synergistic anti-tumor activity when combined with chemotherapy for advanced CRC (93). PDO-sourced transplantation models combine organoid heterogeneity and *in vivo* evaluation, enabling individualized chemotherapy and HER2 targeted therapy screening (94). Humanized models address low immune response in MSS CRC treatment, demonstrating that TGF-β inhibitors combined with PD-1 blockade reverses immunosuppressive TME (70, 95–97). They also validate BRAF-targeted therapy and immunotherapy synergism. Ectopic and metastasis models simulate clinical scenarios like liver metastasis, supporting development of anti-metastasis drugs (such as MET inhibitors).

The use of xenograft tumor animal models for human colorectal cancer precision medicine has limitations: There is no clear systematic correlation with CRC molecular typing (CMS, MSI/MSS), making it difficult to meet the needs of precision medicine for patient heterogeneity and molecular characteristics. It can only simulate the basic growth of tumors and cannot stably reproduce the characteristics of each CMS subtype, such as the high immunogenicity of CMS1 type, the interstitial immunosuppressive microenvironment of CMS4 type, and the stable recurrence of MSI-H mutation features. It also fails to reflect the differences in BRAF/KRAS mutations, immune microenvironment, and treatment responses of patients. Moreover, there are currently no clear systematic requirements for addressing the limitations of this model, making it difficult for the research results to directly serve CRC precision diagnosis and treatment.

These models cover drug screening, efficacy prediction and drug resistance mechanism studies, building a translational bridge from clinical tumor characteristics to molecular targets and precise therapy, thus optimizing individualized CRC treatment strategies.

However, PDX models also have unavoidable limitations: 1) The success rate of modeling is greatly affected by the quality of tumor tissue. The tumor tissue of advanced CRC patients has severe fibrosis and high necrosis rate, and the success rate of modeling is only 40-60%, significantly lower than that of early patients; 2) Tumor heterogeneity may be lost during long-term passage, especially the disappearance of rare subclones; 3) Murine stromal cells may replace human stromal cells, affecting the integrity of the tumor microenvironment.

### 3.4 Spontaneous models

Spontaneous CRC models utilize inbred strain rodents such as aged C57BL/6 mice and WF-Osaka rats—which are genetically predisposed to diseases—to mimic natural pathogenesis of human colorectal cancer without external carcinogens exposure or genetic engineering (98, 99). These models recapitulate key aspects of tumor development and are particularly valuable for studying innate tumorigenesis under controlled genetic backgrounds. For instance, WF-Osaka rats exhibit a tumor incidence rate of 30-40%, making them suitable for basic research on spontaneous tumorigenesis under controlled genetic backgrounds (100–102).

The clinical translation and application value of spontaneous animal models in precision medicine are limited due to several drawbacks: the tumor incidence rate is only 30%-40%, the tumors are randomly distributed in the digestive tract or concentrated in the proximal colon, which does not match the characteristic of FAP in humans where the lesions are concentrated in the colon and rectum. Therefore, it is impossible to verify the efficacy of colon-specific treatment plans; moreover, this model has a large difference from the human genetic background, with an MSI incidence rate lower than 3%, lacking the driver mutation spectrum and subtypes of human CRC, and due to the differences in drug metabolism and immune mechanisms between species, the clinical reference value for predicting treatment responses is limited.

Moreover, their applicability is constrained by low tumor incidence rates (some below 10%), prolonged latency periods (over eight months), unpredictable lesion location and growth patterns, and poor experimental reproducibility. As a result, spontaneous models are unsuitable for high-throughput drug screening or therapeutic evaluation (100, 103), and are primarily limited to basic mechanistic studies that leverage their natural disease progression for pathophysiological insight.

### 3.5 Non-murine models of CRC

Complementing traditional murine systems, non-murine models—including zebrafish, pigs, dogs, and fruit flies—offer distinct advantages for CRC research. These non-murine models enhance experimental tractability, physiological relevance, and translational value, offering complementary insights into CRC pathogenesis, therapeutic response, and TME dynamics.

#### 3.5.1 Zebrafish models

The zebrafish (*Danio rerio*) exhibit genomic homology with humans (70-82%) and conserves numerous disease-related genes and epigenetic regulators (104). Key advantages include high fecundity, rapid embryonic development, and optical transparency during larval stages enabling real-time observation, and efficient genetic manipulation through CRISPR-Cas9 and Tol2 transposon systems. These characteristics support shortened experimental timelines, reduced costs, and enhanced suitability for high-throughput drug screening, making zebrafish as a cost-effective complement to rodents (105). In CRC precision medicine, it takes 3–6 months to establish mouse PDX model (6–8 weeks for drug susceptibility testing), and the molecular and microenvironment are single, which are difficult to connect CMS typing, and there is a gap of “rapid personalized testing”. However, Zebrafish PDX (zPDX) only needs about 100 tumor cells, and can establish the model in 1–2 weeks, complete high-throughput drug sensitivity in 7 days, and observe the inhibition of metastasis in real time, so as to fill the gap in the mouse model and meet the needs of rapid personalized treatment for advanced patients.

Two primary model types are used:

1. Transgenic models utilize tissue-specific promoters to simulate human CRC drivers, revealing tumor heterogeneity and angiogenic-immune crosstalk (106).
2. Xenograft models involve injecting fluorescently labeled human CRC cells into immunodeficient larvae, enabling real-time tracking of metastasis and drug response (107).

In precision medicine applications, PDX zebrafish models allow individualized therapy testing. Genetic models (*e.g.*, APC-deficient models for Wnt pathway, KRAS^G12V^/BRAF^V600E^ mutants) facilitate targeted therapy evaluation (108, 109). ERPP-derived polysaccharides and the triazin sulfonamide MM-129 inhibit molecular targets through signaling cascades in the xenograft models, providing novel approach for chemotherapy of CRC (110–112). However, limitations include gene duplication complicating genetic studies, temperature-dependent cell proliferation differences, and incomplete TME immune recapitulation (113, 114). Future improvements require advanced gene editing and humanized microenvironment.

The zebrafish animal model of colorectal cancer has a genomic homology of 70-82% with the human genome. It is quick to establish and has low cost. The zPDX model can complete drug sensitivity testing during the waiting period for patient treatment, which helps in the research of individualized and targeted therapies for CRC. However, zebrafish have a short lifespan and cannot simulate anatomical location and subtype heterogeneity. There is no clear systematic correlation with molecular typing, and it cannot stably reproduce the characteristics and mutation status of relevant typing. Moreover, there are problems such as gene replication and temperature affecting cell proliferation, and there are no clear norms, making it difficult to fully support precise diagnosis and treatment.

#### 3.5.2 Porcine models

Pigs share 98% genomic similarity with human beings and mirror human anatomy, physiology, and body/organ size, enabling clinical procedures such as colonoscopy (115, 116). They serve as high-fidelity translational bridges between rodents and humans (117). The anatomical structure and intestinal flora of the pig model are 85% similar to those of humans and are highly consistent. The distal colon can be adapted to clinical endoscopy and radiotherapy equipment to simulate minimally invasive/surgical scenarios, and fill the gap in the translational research of minimally invasive and radiotherapy combined therapy for CRC in mice. At the same time, its immune system has 90% homology with human, which can reproduce the adverse reactions related to immunotherapy, which cannot be achieved in mice.

Porcine CRC models include:

1. Spontaneous/induced models: Germline APC mutants mimicking familial adenomatous polyposis, DSS-induced colitis-associated cancer models, and germ-free pigs colonized with human microbiota for studying diet-microbiome interactions (118, 119).
2. Genetically engineered models: such as, APC^1311/+^ line, LGR5-H2BGFP reporters for tracking intestinal stem cells, and the “Oncopig” model with Cre-inducible oncogenic mutations for dynamic progression monitoring (120–122).

Advantages comprise compatibility with clinical equipment, relatively longer lifespan than rodents, human-like immune responses, and biomarker validation utility (123–125). The TME of pig models is highly similar to that of humans in terms of immune cell subset ratio and cytokine profile (such as IL-10 and IFN-γ levels). The number of goblet cells and tight junction structure in the colonic mucosa are consistent with those of human CRC, and can accurately simulate the immunosuppressive TME of MSS tumors (126). Limitations involve high costs, small cohorts, prolonged tumor latency (115, 127, 128). Applications include validate endoscopic ablation techniques, radiotherapy regimens, and chemotherapeutic protocols (116, 120). Future directions focus on miniature breeds (for cost reduction, *etc.*), optimized gene editing (for distal CRC), and integrated “mouse-screen, pig-validation” pipelines for commercial applications (129).

The structure of the colon, the intestinal microbiota, and humans are highly matched. It can be directly used with clinical equipment, precisely simulating the immunosuppressive microenvironment of MSS tumors. There are multiple model types that can verify the treatment plans, filling the gap in the translational research of mouse models. However, this model has problems such as high cost, small cohort size, long tumor latency period, single molecular subtype, insufficient high-throughput analysis, and there is no clear specification for addressing its limitations, making it difficult to fully support precise CRC diagnosis and treatment.

#### 3.5.3 Canine models

Pet dogs (*Canis lupus familiaris*) develop spontaneous CRC with similarities to humans in size, physiology, and environmental exposures (130). Incidence is below 1%, but breed predisposition can exist. Tumors often involve Wnt pathway dysregulation and progress from adenomas to adenocarcinomas in distal colon/rectum (131).

These models enable metastatic studies and align with clinical imaging and treatment protocols (132). Their immune resemblance supports the evaluation of immunotherapies and NSAIDs (133, 134). Limitations include low incidence rates, cohort assembly challenges, and inability to induce tumors experimentally (135). Dogs contribute to pharmacokinetic and metastasis researches via clinical trial participation (136). Future efforts require multi-institutional molecular profiling, canine PDX models, and breeding of susceptible lineages (137, 138).

The canine model of colorectal cancer can spontaneously develop CRC, with tumors and physiological conditions similar to those in humans, which can assist in diagnosis and treatment research. However, its incidence is low, the construction of the cohort is difficult, and the matching degree of molecular subtypes is low. Species differences limit its clinical reference value.

#### 3.5.4 Drosophila models

The fruit fly (*Drosophila melanogaster)* serves as an invertebrate model for CRC research, by leveraging conserved signaling pathways (Wnt, RAS/MAPK, and JNK, *etc.*) for CRC mechanistic studies (139). Its midgut contains regenerative stem cells (140, 141), and models multi-step carcinogenesis when APC deletion combines with RAS activation and polarity gene deletion (142, 143).

Strengths include powerful genetic tools for large-scale screens, rapid generation of complex genotypes, and feasibility for small-scale drug testing (143–145). Limitations arise from evolutionary distance, missing human CRC features, and incomplete gene conservation. *Drosophila* aids target identification and preliminary drug assessment in precision oncology.

The fruit fly model can be used to study the mechanisms of CRC, simulate carcinogenesis, and assist in the research of drugs and targets. However, it can only simulate the basic carcinogenic pathways and cannot reproduce heterogeneity and the immune microenvironment. It has a weak correlation with CRC molecular typing and shows significant differences from humans, making it difficult to support precise diagnosis and treatment of CRC.

### 3.6 Modeling CRC risk factors

Accumulating clinical evidence implicates chronic intestinal inflammation, high-fat diet (HFD), and pathogenic gut microbiota as of CRC risk factors (146). These are recapitulated in laboratory animal models, where they may accelerate tumorigenesis in genetically predisposed backgrounds (147, 148).

#### 3.6.1 Inflammation-associated CRC models

Chronic inflammation elevates CRC risk, particularly in inflammatory bowel diseases (IBD) such as ulcerative colitis and Crohn’s disease (149). The DSS model induces ulcerative colitis like injury, mostly characterized by bloody stools, ulceration, and granulocyte infiltration, closely mimicking human ulcerative colitis (150, 151). When combined with AOM, it shortens tumor development from 10–20 weeks to 6–10 weeks. This AOM/DSS model reproduces distal colon tumors with progressive pathology (adenomas to adenocarcinomas), enabling study of dietary, pharmaceutical, and microbial influences on inflammation-driven cancer (152). However, the AOM/DSS model has significant limitations that seriously affect its clinical translation value: 1) Low genomic fidelity: Its tumor mutation spectrum is dominated by inflammation-related mutations (such as TP53 R248W), lacking common hot-spot mutations of APC and KRAS in human CRC (APC^T876^, KRAS^G12D^), and the mutation coincidence rate is only 40%-50%; 2) Random tumor occurrence: The number of tumors and size in the same batch of models vary greatly, resulting in an experimental reproducibility variation coefficient of 25%-30%; 3) Weak metastatic ability: Only less than 5% of models will have local lymph node metastasis, unable to simulate the liver and lung metastasis characteristics of human CRC (clinical metastasis rate 30%-40%).

#### 3.6.2 High-fat diet models

HFD with >30% fat promote metabolic and inflammatory changes linked to CRC (153) (154),. Models identify therapeutic targets (*e.g.*, TLR4/CXCL10, STAT3, and GPR65) and validate agents like evodiamine and naringenin that regulate inflammation, EMT, and microbiome balance. HFD models also reveal immunosuppressive microenvironments and test combinational therapies (*e.g.*, anti-PD-L1 antibodies with CSF-1R inhibitors) (155–158). The microbiota and metabolism exert their effects through intestinal microbiota modulation, bile acid metabolism, or tumor energy supply, expanding the scope of non-pharmacological precision interventions (159). Synergy of short-term HFD and PD-L1 antibody, enhanced by the addition of CSF-1R inhibitor and chemotherapy, addresses challenges related to HFD-induced immunosuppressive microenvironments in CRC and chemotherapy resistance (160, 161). These strategies provide a platform for precision intervention spanning target validation to combination strategy optimization.

#### 3.6.3 Gut microbiota in CRC

Gut microbiota has emerged as a critical environmental factor in CRC initiation and progression, influencing tumor development through complex host-microbial interactions. Specific microorganisms—including *Fusobacterium nucleatum*, enterotoxigenic *Bacteroides fragilis*, *Porphyromonas gingivalis*, and others—correlate with CRC recurrence, metastasis, and poor prognosis (162). These interactions can be modeled via fecal microbiota transplantation in genetically engineered or chemically induced CRC models.

*F. nucleatum* promotes metastasis in APC^Min/+^ mice through autophagy activation and modulates the tumor microenvironment via CCL20-mediated macrophage recruitment and M2 polarization. Notably, it may enhance anti-PD-1 efficacy in microsatellite-stable CRC by increasing butyric acid and reducing CD8^+^T cell exhaustion (163, 164). *Parvimonas micra*, an oral pathobiont, colonizes the colon via the oral-gut axis, upregulates Cyclin D1, inhibits p53 signaling, and activates NF-κB through Th17 cell induction. *Porphyromonas gingivali* correlates with reduced overall survival in CRC patients and accelerates tumorigenesis in APC^Min/+^ mice by recruiting tumor-infiltrating myeloid cells, activating the NLRP3 inflammasome, and increasing tumor burden (165).

Probiotics such as *Lactobacillus gallinarum* and *Lactobacillus plantarum* enhance immunotherapy response through metabolic modulation of the IDO1/Kyn/AHR axis and CD8^+^ T cell activation (166). Potential mechanisms include competitive exclusion of pathogens, production of short-chain fatty acids, reinforcement of the intestinal barrier, and improved chemotherapy tolerance, highlighting the microbiome as a biomarker and therapeutic target for CRC treatment.

Inflammatory, hyperlipidemic, and microbial animal models can respectively simulate the occurrence of CRC triggered by a single pathogenic factor, each having its own research value. However, they also have significant limitations: they can only simulate a single pathogenic factor, deviating from human diseases and classifications, and have low genomic fidelity.

## 4 Challenges of animal models in meeting precision medicine requirements for CRC

Precision medicine for CRC requires models that heterogeneity and treatment response. Current animal models face significant challenges in replicating key clinical, molecular, and immunological features of human CRC, limiting their utility in biomarker discovery and therapeutic development for clinical transformation (**Tables 4**, **5**).

**Table 4:** Strengths, limitations, and precision medicine applications of major CRC development models.

Animal model	Advantages	Disadvantage	Precision treatment application	References
Carcinogen-Induced Models	Recapitulates the inflammatory-carcinogenesis process of human CRC	Long experimental duration, significant inter-individual variability, difficulty in monitoring tumor size, and potential systemic toxicity.	Identification of inflammation-related pathogenic targets; evaluation of chemopreventive agents; validation of progression blockers.	(32, 100, 208)
Genetically Modified Model	Suitable for investigating genes implicated in colorectal cancer and for immunotherapy research.	Tumors exhibit locations and pathological features significantly different from those in humans. Low tumor incidence, high cost, and long experimental duration.	Screening for driver genes amenable to targeted therapy; testing gene-editing therapies; evaluating mutation-specific immunotherapy combinations.	(32, 209)
Transplantation Models	High success rate, rapid tumor formation, short experimental cycle, suitable for immunotherapy and drug screening.	Unable to recapitulate the early stages of tumorigenesis; dependent on the characteristics of tumor cell lines and host animals.	High-throughput precision drug screening.	(210)
Spontaneous Models	Tumors possess clinical and genetic features similar to human CRC, reflecting the natural process of tumor development.	Limited case availability, significant inter-individual variability, high husbandry and experimental costs.	Translating preclinical findings; evaluating personalized therapies; studying tumor heterogeneity in treatment responses.	(100)
CRC Risk Factor Models	Useful for simulating the role of risk factors (*e.g.*, obesity) in CRC and exploring the mechanistic links between metabolic disorders and CRC.	Lengthy model establishment period; obesity-induced tumor phenotypes may be atypical; presence of multiple confounding variables.	Developing risk factor-related preventive strategies; evaluating metabolism-targeted personalized therapies; studying microbiota-modulated treatments.	(87, 155)

**Table 5:** Comparative analysis of multi-species animal models for CRC research: strengths, limitations, and precision oncology applications.

Species	Advantages	Disadvantage	Precision treatment application	References
Mouse (*Mus* *musculus*)	High (~95%) genetic homology to human; recapitulates key CRC features; genetically tractable; rapid breeding	Species-specific immune/metabolic/gut microbiome differences	Facilitates testing of targeted therapies (*e.g.*, EGFR inhibitors, immune checkpoint inhibitors) and subtype-specific drugs (*e.g*., iNOS & KRAS inhibitors)	(34, 211)
Zebrafish (*Danio rerio*)	Rapid reproduction; transparent embryos enable real-time observation; ideal for high-throughput drug screening and studying early tumor angiogenesis/metastasis	Marked physiological differences from humans; simple adult intestinal structure; limited simulation of complex tumor microenvironment and immune response	High-throughput drug screening; personalized therapy prediction using PDXs (avatars); investigating anti-angiogenic treatments.	(113, 114)
Pig (*Sus* *scrofa*)	High intestinal anatomical/physiological similarity to humans; suitable for simulating CRC surgery, chemo-radiotherapy responses, and tumor microenvironment studies	High breeding costs and long life cycle; limited application of gene-editing technologies; ethical considerations.	Validating precision surgical techniques; evaluating personalized chemo-radiotherapy regimens; investigating microenvironment-targeted therapies.	(116, 120)
Dog (*Canis* *familiaris*)	Spontaneous CRC mimics human clinical phenotypes/genetics; aids tumor immune microenvironment research; high translational value	Limited cases, marked individual variability; high husbandry/experimental costs; genetic manipulation challenges	Translating precision immunotherapy findings; evaluating advanced personalized CRC protocols; studying immune-modulated treatments	(135, 212)
Drosophila (*Fruit fly*)	High human genetic conservation, easy genetic manipulation; rapid reproduction, short lifecycle; ideal for high-throughput genetic screening	Simplified intestinal structure, marked divergence from human CRC pathology; failure to simulate tumor microenvironment complexity	Precision therapy target screening; predicting personalized chemo-targeted combinations; studying conserved signaling pathways	(143, 144, 213)

### 4.1 Anatomical and genetic relevance

Most current transgenic CRC models develop tumors primarily in the small intestine, contrasting with the predominant rectal and colonic localization in humans. The anatomical mismatch between models and human CRC not only affects the quantitative evaluation of treatment effects but also may lead to biomarker misjudgment. For example, the proportion of MSI-H subtypes in small intestine-derived tumor models is about 25%-30%, which is significantly higher than that in human distal CRC (12%-15%). If drugs targeting MSI-H are screened based on this model, the efficacy may be lower than expected during clinical translation. Furthermore, commonly used mutation sites (*e.g.*, APC^T850^) often differ from clinically prevalent mutations (*e.g.*, APC^T876,T1309,T1450^) (167), reducing translational relevance. Combining genetic engineering with chemical induction (*e.g.*, AOM/DSS treatment in APC^Min/+^ mice) or employing large animal models such as pigs may better mimic pathogenesis and improve metastatic modeling, representing a promising future direction.

### 4.2 Molecular and immunological annotation

Although some models have shown responses to PD-1 inhibitors and anti-EGFR therapies in the studies, the validation standards for MSI and EGFR status in different laboratories are inconsistent - for instance, some studies did not verify the expression of MMR proteins through IHC, but relied solely on PCR to detect the stability of microsatellite sites, resulting in reduced comparability of the results. Therefore, the current molecular characterization of the models faces the challenge of lack of a unified standard, rather than being completely lacking in annotations. For another example, although the AOM/DSS model responds to immune checkpoint inhibitors like anti-PD-1 or anti-EGFR therapy, its MSI or EGFR status often remains unverified, complicating mechanistic interpretation (28, 29). Enhanced genomic, transcriptomic, and proteomic profiling, along with established model databases, would strengthen validity and reproducibility.

### 4.3 Personalized immunocompetent models

**Figure 3:**
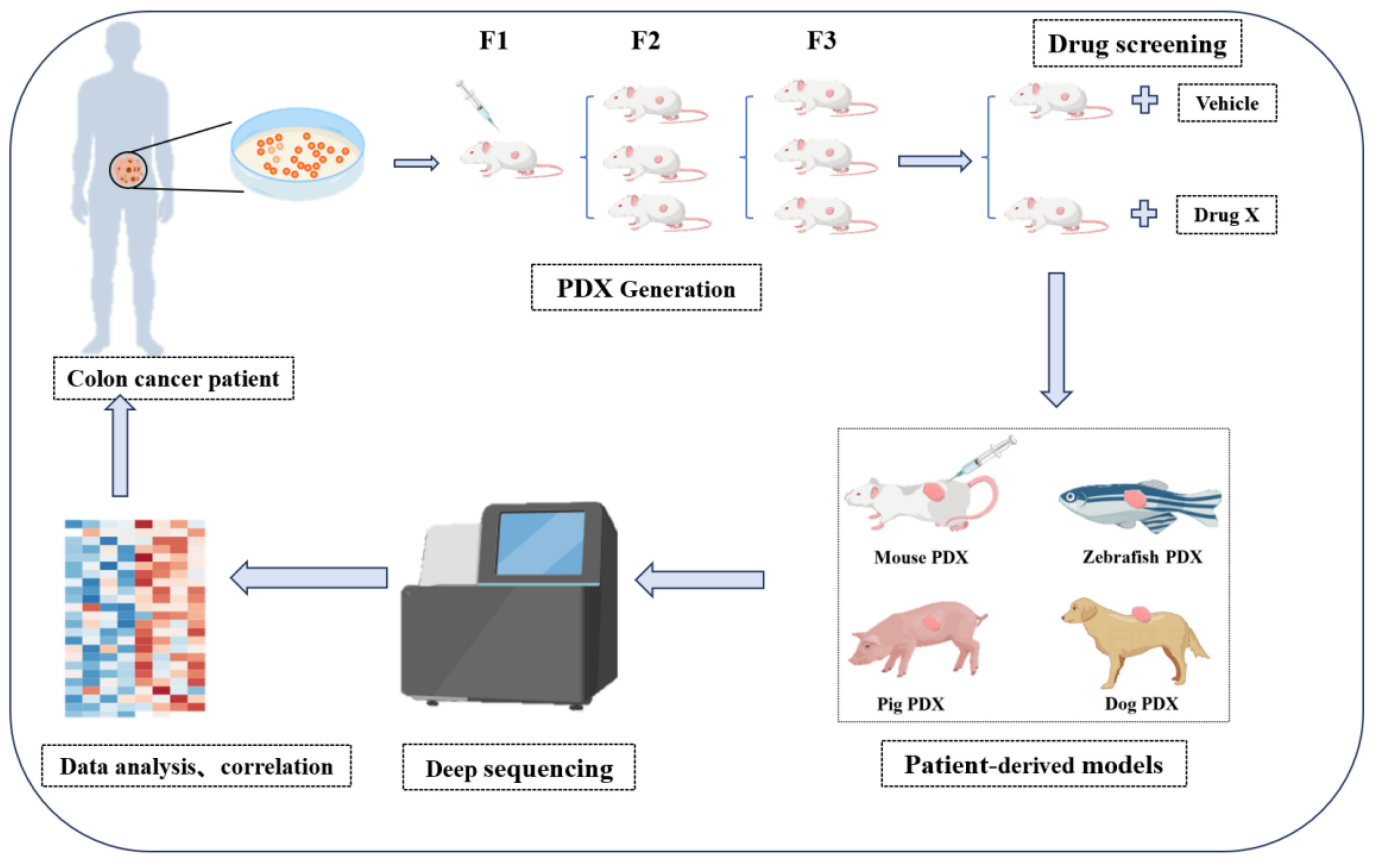
Schematic of PDX models for CRC research. Tumors from CRC patients are engrafted into immunodeficient mice to establish PDX models (spanning F1 to F3 generations). These models were subsequently used for *in vivo* drug screening. The integration of various patient-derived models and deep sequencing-based data analysis facilitates the correlation of preclinical results with individual patient profiles, supporting the development of personalized therapeutic strategies.

While PDOs and PDXs enable drug sensitivity testing, they typically lack functional immune microenvironment. Humanized models, generated via hematopoietic stem cell transplantation, offer partial solutions but face technical challenges including low engraftment efficiency and extended timelines. Alternative approaches, such as immunocompetent PDXs in humanized pigs, may provide more physiologically relevant platforms for immunotherapy evaluation (**Figure 3**).

### 4.4 High-throughput *in vivo* platforms

Conventional PDX models require prolonged serial passaging, delaying drug testing rather than using PDO models. zPDX system overcome this bottleneck by using minimal cell numbers (approximately 100 cells) and leveraging rapid embryonic development, enabling rapid *in vivo* drug sensitivity assays. Emerging clinical evidence suggests zPDX-guided therapies may improve disease-free survival in CRC patients, supporting its potential in clinical decision-making and preclinical trials (105, 168–170).

### 4.5 Expanding therapeutic targeting

**Figure 4:**
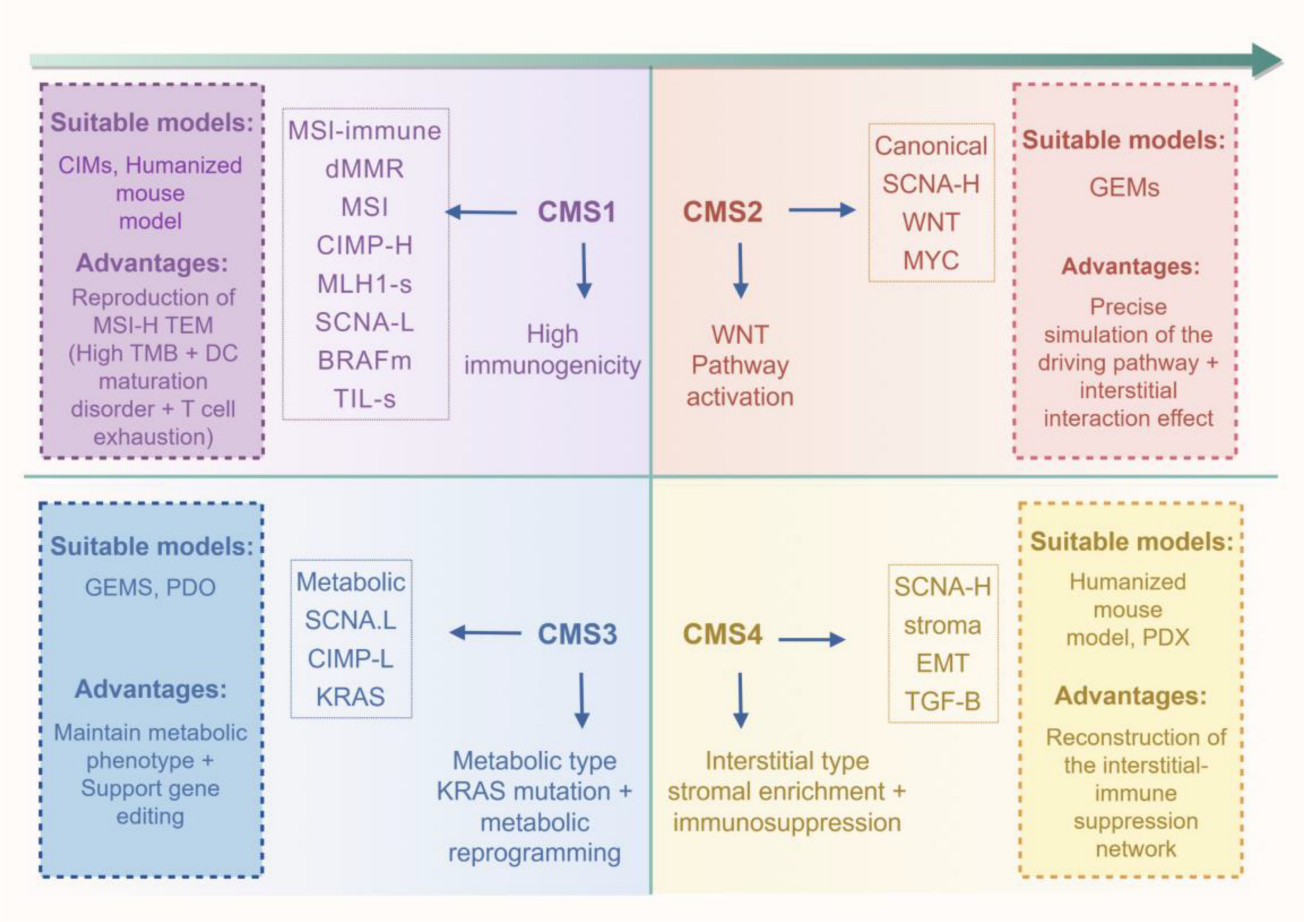
Selecting preclinical models for CRC consensus molecular subtypes. This schematic aligns the four CMS of CRC with their recommended preclinical models, based on shared biological features. CMS1 (immune), defined by dMMR/MSI status and high immunogenicity, is best modeled using CIMs or humanized mice to recapitulate its immune-active tumor microenvironment. CMS2 (canonical), driven by WNT pathway activation, is most accurately simulated with GEM models. CMS3 (metabolic), associated with KRAS mutations and metabolic reprogramming, can be effectively studied using GEMs or PDOs to maintain its distinct metabolic phenotype. CMS4 (mesenchymal), characterized by an EMT-like signature and immunosuppressive stroma, is suitably modeled in PDX systems to reconstruct its complex tumor-stromal-immune interactions. This framework provides a rationale for selecting context-specific models in precision oncology research. (Created with Figdraw.com).

**Figure 5:**
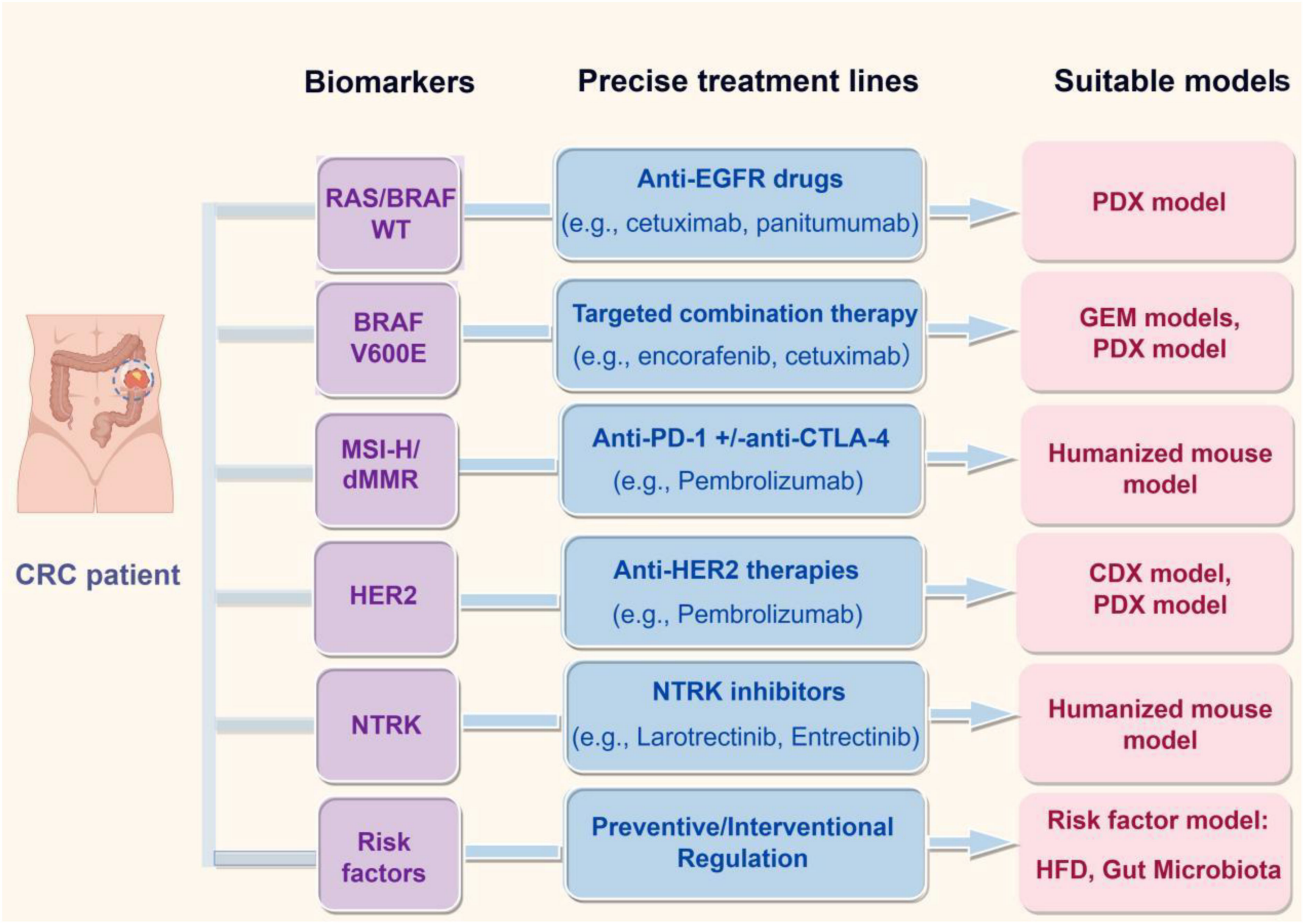
Biomarker-guided precision treatment matching and corresponding preclinical models for CRC. This schematic presents a decision framework for personalized CRC management, aligning patient-specific biomarkers with optimal therapeutic strategies and preclinical validation platforms. The diagram links key molecular determinants—including RAS/BRAF wild-type status, BRAF^V600E^ mutation, MSI-H/dMMR, HER2 amplification, and NTRK fusions—to their respective precision therapies (e.g., anti-EGFR antibodies, targeted combinations, immune checkpoint inhibitors). Corresponding preclinical models, such as PDX, GEMs, humanized mice, and risk-factor-driven systems, are recommended for evaluating each treatment modality, thereby establishing a direct translational bridge from molecular profiling to clinical strategy. (Created with Figdraw.com).

Current targeted therapy for CRC address only a narrow subset of biomarkers *(e.g.*, MMR/MSI, RAS, BRAF, and HER2). Broader therapeutic strategies are needed, particularly for commonly altered genes such as APC, which remains undrugged due to its large protein size and mutational complexity (167). Developing models that better represent diverse molecular subtypes can accelerate novel target identification and validation (**Figures 4**, **5**).

These challenges highlight the need for more clinically aligned, molecularly annotated, and immunologically functional animal models to advance CRC precision medicine.

### 4.6 Limited biomarker-guided model selection

Currently, only a limited number of animal models are well-clarified for studying specific molecular subtypes of CRC. For investigating immunotherapy resistance in MSS tumors, PDX models are preferred due to their ability to recapitulate key immunosuppressive features such as Treg enrichment and elevated PD-L1 expression, making them suitable for probing resistance mechanisms mediated by pathways like TGF-β and IL-6. In cases of MSS tumors with concurrent BRAF^V600E^ mutation, CDX or BRAF-mutant PDX models are recommended for evaluating combination therapies targeting BRAF and EGFR. For the 2–3% of CRC cases characterized by HER2 amplification, PDX or CDX models that retain HER2 amplification (*e.g.*, HER2 copy numbers ≥6) are appropriate for assessing agents like trastuzumab and DS-8201; humanized PDX models further enable the evaluation of combining HER2-targeted therapy with immunotherapy, achieving synergistic inhibition rates of 65–70%. For the more common KRAS-mutant subtypes (~40% incidence), GEM models or relevant PDX models can model MAPK pathway activation and are useful for testing combinations of MEK inhibitors with immunotherapy, showing combined inhibition rates of 45–50%. These examples underscore that model availability remains constrained for many CRC molecular subtypes, highlighting a critical gap in precision oncology research.

### 4.7 Challenges in metastatic fidelity and immune repertoire compatibility

Most preclinical models fail to faithfully recapitulate the metastatic progression of human CRC. A primary limitation is the discrepancy in metastatic patterns: GEM models typically exhibit metastasis rates below 5%, with dissemination primarily to local lymph nodes. This contrasts sharply with the clinical reality in patients, where liver (50%–60%) and lung (20%–30%) are the most common sites of distant metastasis (171). Furthermore, the TME of metastatic lesions in these models often differs from that in humans; for instance, CD8^+^ T cell infiltration in model metastases tends to be higher than the 15%–20% typically observed in human metastatic sites.

These models are further constrained by a fundamental mismatch in immune repertoire between mice and humans. The diversity of the human TCR repertoire (~10^12^) vastly exceeds that of mice (~10^8^), limiting the spectrum of simulated immune responses. Additionally, key differences exist in antibody gene rearrangement, particularly in the diversity of the CDR3 region. These inherent immunological disparities compromise the ability of current models to accurately predict human-specific responses to immunotherapy and to study associated resistance mechanisms (**Table 6**).

**Table 6:** Comparative overview of preclinical models for immuno-oncology research in CRC.

Model type	Immune competence	Mutational fidelity	MSI/MSS representation	Throughput	Cost	Immunotherapy suitability	Core advantages	Key limitations
Chemical Induced	Low (murine-specific)	Moderate (60%-70%)	Partial (MSI-L biased)	Moderate	Low	Inflammation-driven therapies (limited)	Suitable for inflammation-cancer transition research	Unable to reproduce immune desert TME
Genetically Engineered	Low (murine immune)	High (85%-90%)	Partial (CMS2/4 biased)	Low	High	Combined evaluation of targeted therapy and immunotherapy	Can simulate tumorigenesis of specific gene mutations	Lack of human MHC molecules
Cell-line-derived Xenografts (CDX)	Low (immunodeficient host)	Moderate (70%-75%)	Complete	High	Low	Preliminary drug screening (limited TME relevance)	High throughput, low cost	Lack of patient-specific TME
Patient-derived Xenografts (PDX)	Moderate (retains patient TME)	High (90%-95%)	Complete	Low	High	Biomarker-driven personalized therapy evaluation	Retains tumor molecular and TME heterogeneity	Long modeling cycle (3–6 months)
Humanized PDX	High (reconstructs human immune)	High (90%-95%)	Complete	Low	Very High	Immune checkpoint inhibitors, CAR-T, bispecific antibodies	Closest to human clinical scenarios	Low immune reconstitution efficiency
Zebrafish zPDX	Low (larval immune immature)	Moderate (65%-70%)	Complete	Very High	Low	High-throughput drug sensitivity testing	Short modeling cycle, low sample demand	Unable to reproduce immune cell-mediated response
*Porcine*	High (human-like immune)	High (85%-90%)	Complete	Low	Very High	Combined verification of surgery/radiotherapy and immunotherapy	Highly homologous anatomy and immunity with humans	High cost, limited sample size

## 5 Conclusion and future perspectives

Animal models remain indispensable for advancing CRC precision medicine, yet their clinical relevance is limited by anatomical, molecular, and immunological disparities. Future efforts should focus on developing integrated models that incorporate human-relevant genetic mutations, physiologically accurate microenvironments, and functional immune components. The expanded use of multi-omics profiling and non-murine systems—such as porcine or zebrafish-based platforms—can improve molecular annotation and experimental throughput. By closing these translational gaps, next-generation models will accelerate the discovery of biomarkers and tailored therapies, thereby enhancing personalized treatment strategies for CRC patients, ultimately.
